# Clinical evaluation of radiation-induced sinusitis by MRI-based scoring system in nasopharyngeal carcinoma patients

**DOI:** 10.1186/s13014-023-02331-3

**Published:** 2023-09-18

**Authors:** Wenya Zheng, Tao Yan, Dongjiao Liu, Geng Chen, Yingjuan Wen, Xiuli Rao, Yizhe Wang, Huijuan Zheng, Jiahong Yang, Hua Peng

**Affiliations:** 1 Department of Otolaryngology, Head and Neck Surgery, General Hospital of Southern Theatre Command of PLA, Guangzhou, 510010 China; 2 The First School of Clinical Medicine, Southern Medical University, Guangzhou, 510515 China; 3 Department of Cardiology, General Hospital of Southern Theatre Command of PLA, Guangzhou, 510010 China; 4 Department of Medical Imaging, General Hospital of Southern Theatre Command of PLA, Guangzhou, 510010 China; 5 Department of Radiation Oncology, General Hospital of Southern Theatre Command of PLA, Guangzhou, 510010 China

**Keywords:** MRI, Radiation-induced sinusitis, NPC, Evaluation, Risk factor

## Abstract

**Objective:**

To explore the application of magnetic resonance imaging (MRI) in the evaluation of radiation-induced sinusitis (RIS), MRI-based scoring system was used to evaluate the development regularity, characteristics and influencing factors of RIS in nasopharyngeal carcinoma (NPC) patients.

**Patients and methods:**

A retrospective analysis was performed by collecting the clinical and MRI data of 346 NPC patients to analyze the characteristics and prognosis of RIS. The predictive model was constructed according to the influencing factors of RIS.

**Results:**

**(1)** In the 2-year follow-up after radiotherapy (RT), there was significant change in L-M score in both groups of NPC patients (sinusitis before RT group: *p* = 0.000 vs. non-sinusitis before RT group: *p* = 0.000). After 6 months of RT, the L-M scores of the two groups tended to plateau (sinusitis before RT group: *p* = 0.311 vs. non-sinusitis before RT group: *p* = 0.469). **(2)** The prevalence of sinusitis in two groups of NPC patients (without or with sinusitis before RT) was 83% vs. 93%, 91% vs. 99%, 94% vs. 98% at 1, 6 and 24 months after RT, respectively. **(3)** In the patients without sinusitis before RT, the incidence of sinusitis in maxillary and anterior/posterior ethmoid, sphenoid and frontal sinuses was 87.1%, 90.0%/87.1%, 49.5%, 11.8% respectively, 1 month after RT. **(4)** A regression model was established according to the univariate and multivariate analysis of the factors related to RIS (smoking history: *p* = 0.000, time after RT: *p* = 0.008 and TNM staging: *p* = 0.040).

**Conclusion:**

**(1)** RIS is a common complication in NPC patients after RT. This disorder progressed within 6 months after RT, stabilized and persisted within 6 months to 2 years. There is a high incidence of maxillary sinus and ethmoid sinus inflammation in NPC patients after RT. **(2)** Smoking history, time after RT and TNM staging were significant independent risk factors for RIS. **(3)** The intervention of the risk factors in the model may prevent or reduce the occurrence of RIS in NPC patients.

## Introduction

NPC, with obvious geographical distribution, is particularly prevalent in East and Southeast Asia, as well as in eastern North Africa, Greenland and Alaska. In China, NPC is most common in the central and western regions of Guangdong Province [1]. Studies have shown that the incidence of NPC in 2019 was 45.09% higher than that in 2009, and the age-standardized incidence rate (ASIR) increased from 1.81 to 2009 to 2.12 in 2019 [2].

While the age-standardized death rate (0.93 in 2009 and 0.86 in 2019, respectively) and the age-standardized disability-adjusted life year rate (30.22% in 2009 and 27.98% in 2019, respectively) showed a decreasing trend [2]. Several studies have reported a slight worldwide downward trend in incidence and mortality of NPC [1, 3]. NPC is characterized by rapid local invasion, early lymphatic spread and distant metastasis [4].

Due to the highly sensitivity to ionizing radiation of NPC, RT or RT-based comprehensive treatment is its main treatment [5]. With the development of radiation techniques, RT for NPC has developed from traditional two-dimensional radiation therapy (2D-RT) to three-dimensional conformal radiation therapy (3D-CRT), followed by advanced intensity-modulated radiation therapy (IMRT) and stereotactic radiation therapy (SRT). Helical tomotherapy (HT) integrates IMRT, image-guided radiation therapy (IGRT) and dose-guided radiation therapy (DGRT). It combines diagnostic radiology and RT so can accurately target all kinds of lesions, especially for NPC with irregular target region and similar critical adjacent structures [6, 7]. After receiving IMRT, more than 90% of NPC patients achieved excellent local-regional control and the 5-year regional failure-free survival rates of N_0 − 1_ and N_2 − 3_ patients were 98.5% and 90.2%, respectively [8]. For newly diagnosed, biopsy-proven and non-metastatic NPC patients, IMRT reduced the incidence of 5-year locoregional failure to 7.4% [9]. RT combined with chemotherapy or immunotherapy showed a better locoregional control than RT alone, resulting in longer progression-free survival and overall survival for patients with locoregionally advanced NPC. The comprehensive treatment also helped patients with recurrent or distant metastasis of NPC benefit more [10–12]. As survival rate has improved, more attention has been paid to the toxicity and side effects of RT with or without chemotherapy in patients with NPC and the quality of life of these patients [13].

RT or chemoradiotherapy (CRT) for NPC is unexpectedly accompanied by a variety of acute and chronic complications, such as radioactive otitis media, radiation encephalopathy, osteoradionecrosis, radiation dermatitis and so on. The prevalence of post-radiation sinusitis is high in postirradiation NPC patients, who often presented with symptoms of nasal congestion, purulent rhinorrhea, headache and other symptoms. These symptoms cause sleeping problems, depression, fatigue, anxiety and some psychological problems. Due to radiation damage of nasal mucous membrane, post-radiation sinusitis commonly has more severe symptoms than other types of rhinosinusitis [14]. Besides, the effect of clinical treatment is worse than that of common sinusitis, which seriously affects the long-term quality of life of the NPC patients [14–16]. However, at present there is still a lack of research on the post-radiation sinusitis, one of the toxic and side effects of different RT or CRT regimens on patients with NPC. Moreover, the clinical observation and evaluation of sinusitis characteristics after HT or 3D-CRT by MRI has rarely been reported in the literature [17]. The purpose of this study was to evaluate the prevalence, clinical features and prognosis of post-radiation sinusitis in NPC patients by MRI.

The paranasal sinuses are air-filled cavities within the skull. Because of its strong ability to distinguish the deep tissue, imaging has become an important examination to evaluate the location and severity of sinonasal lesions. Among the imaging examinations, computer tomography (CT) scan is preferred to evaluate the acute and chronic nasal inflammation [18]. However, as NPC patients are often revisited by oncologists, both of the patients and doctors usually tend to pay more attention to the tumor than sinusitis. Furthermore, repeated CT scans are potentially harmful due to cumulative radiation exposure. Therefore, although there is a very high prevalence of radiological sinusitis in NPC patients after RT, CT examination is rarely taken as evaluated method. Most imaging studies of sinusitis are based on CT scoring system, but MRI is superior to CT in distinguishing inflammatory lesions from neoplastic lesions and in evaluating sinusitis intracranial complications [19–21]. Hence, pretreatment clinical stage and regular re-examination were usually assessed by head and neck MRI to evaluate the tumor status [22]. Therefore, whether MRI can substitute for CT to evaluate post-radiation sinusitis is of great practical value for patients with NPC. Some studies have shown that there was a good correlation between CT and MRI scores when using Lund-Mackay (L-M) scoring system to evaluate sinusitis. There was no significant over-grading and misjudgment of sinusitis on MRI, and a significant correlation between MRI performance and sinusitis symptoms [23, 24]. For these reasons, MRI may be an effective method to evaluate post-radiation sinusitis.

Consequently, we conducted a retrospective study based on the MRI scoring system to explore the clinical characteristics and risk factors of sinusitis before and after 3D-CRT or HT on NPC. The main objective of this study was to provide evidence for the MRI-based evaluation of sinusitis after different RT modalities on NPC, distinguish its clinical characteristics and risk factors, as well as establish a predictive MRI-based radiomics nomogram and evaluate the predictive ability of the model.

## Materials and methods

### Study population

This study was a retrospective analysis. Ethical approval was obtained from the Ethics Committee of General Hospital of Southern Theatre Command of PLA for the study. Inclusion criteria: Patients with newly-diagnosed, histologically-proven NPC and MRI radiographic records before and after RT in the General Hospital of Southern Theatre Command of PLA (formerly known as General Hospital of Guangzhou Military Command) from January 2015 to January 2019 were included. Exclusion criteria: Patients whose medical record or RT regimen information was incomplete were excluded. The study population was divided into two groups: (i) group A: patients with sinusitis before RT and (ii) group B: patients without sinusitis before RT. This study was approved by the ethics committee of the General Hospital of Southern Theatre Command of PLA.

### Clinical stage

All patients were evaluated before treatment (complete medical history, physical examination, hematological and biochemical examination, nasopharynx and neck MRI, chest CT, abdominal ultrasonography and whole-body bone scan). Reclassification was performed in all patients according to the 8th edition of the Union for International Cancer Control/American Joint Committee on Cancer (UICC/AJCC) staging system.

### Radiotherapy

Three hundred and forty-six patients with NPC who received 3D-CRT or HT in the hospital were included in our study, of which 145 patients received 3D-CRT and 201 patients received HT. In order to treat the primary tumor and the upper neck region above the superior border of the clavicle, all patients were treated with 6–10 MV photons from linear accelerators (3D-CRT:Varian Co., HT:CNNC Accuray Co.) and patients who received 3D-CRT were also given 6‑16 MeV clinical linear accelerator (Varian Co.) electron beam.According to the patient’s wish and condition, a personalized treatment plan for each patient was developed including RT technology and fractionated dose by oncologists. The gross tumor volume (GTV) included the gross primary tumors (GTVnx) and involved lymph nodes (GTVnd) determined by clinical and imaging examinations. The clinical target volume (CTV) consisted of CTV1 and CTV2. CTV1 was to encompassed the whole nasopharynx, retropharyngeal lymph nodal regions and any high-risk nodal region (usually the GTVnx plus a 6 to 8 mm margin). CTV2 was defined as CTV1 plus a 6 to 8 mm margin, as well as GTVnd and cervical lymph node drainage regions requiring prophylactic irradiation. The GTVnx plus a 3 mm margin was defined as planning gross tumor volume (PGTVnx) performed with 64–76 Gy and the GTVnd plus a 5 mm margin was defined as PGTVnd performed with 60–70 Gy. PTV1 was defined as CTV1 plus a 3 mm (HT) / 5 mm (3D-CRT) margin performed with 60 Gy and PTV2 was defined as CTV2 plus a 3 mm (HT) / 5 mm (3D-CRT) margin performed with 54 (HT)/50 (3D-CRT) Gy.

The conformity index (CI) and the homogeneity index (HI) of the target area are considered as two main factors to evaluate the radiotherapy plan. CI=(TVRI/TV) × (TVRI/VRI) (TVRI: the volume of the target area included in the reference isodose line; TV: the total volume of the target area; VRI: the volume of the area included in the reference isodose line). The value of CI ranges from 0 to 1. The closer to 1 the CI value is, the better the conformity of the target area is. HI = D5%/D95% (D5%: the dose received by 5% of the volume in the target area; D95%: the dose received by 95% of the volume in the target area). During HT treatment, the patient was placed according to the position of the simulated CT position, and then underwent a CT scan. Bone markers were used to make these CT images matching the CT images at the time of positioning. The verification could be passed if the error was less than 3 mm. While during 3D-CRT treatment, x-ray was used for verification with the error value less than 5 mm. And then x -rays were taken again under the linear accelerator for verification. Compared with 3D-CRT, the HT technology was superior in both target area CI and HI. That is, HT makes the target area achieving the prescribed dose while minimizing the radiation dose of spinal cord and brainstem, which was obviously beneficial for protecting the central nervous system. NPC patients received RT with 1.68–2.18 Gy/time in HI treatment or 2 Gy/time in 3D-CRT treatment, five times a week, once a day, and 32 to 33 times (HT) or 35 treat times (3D-CRT) in total.

### Chemotherapy

During the study period, institutional guidelines recommended RT alone for stage I (T1N0M0) and some of stage II (T2N0M0), concurrent chemoradiotherapy (CCRT) +/- induction chemotherapy or adjuvant chemotherapy for stage II (except T2N0M0) to stage IVa, and platinum-based chemotherapy +/- CCRT + additional RT or chemotherapy according to clinical indications for stage IVb. Overall, 10 patients (2.9%) received RT alone, and 336 patients (97.1%) received chemotherapy and RT. Concurrent chemotherapy consisted of either cisplatin or nedaplatin given once a week (30 mg/m^2^) or on the 1st, 22nd and 43rd days of RT(40 mg/m^2^). The total courses of 3D-CRT or HT was 49 or 44 days, respectively. The most common induction / adjuvant chemotherapy regimens were TPF (paclitaxel 210 mg/m^2^ on day 1, platinum 40 mg/m^2^/day on day 1–3, 5-fluorouracil 750 mg/m^2^/day on day 1–3), TP (paclitaxel 210 mg/m^2^ on day 1, platinum 40 mg/m^2^/day on day 1–3), DP (docetaxel 75 mg/m^2^ on day 1, platinum 75 mg/m^2^ on day 1) and DPF (docetaxel 75 mg/m^2^ on day 1, platinum 75 mg/m^2^ on day 1, 5-Fluorouracil 500 mg/m^2^/day on days 1–5). Which specific chemotherapy scheme was left to the discretion of the treating physician.

### MRI

The region from the suprasellar cistern to the inferior margin at the sternal end of the clavicle was examined in all patients before treatment and 1, 3, 6, 9, 12, 18 and 24 months after RT on a 3.0-Tesla superconductive MRI system (Signa HDxt, General Electric Healthcare, USA) using an eight-channel head and neck combined coil in supine position. T1-weighted axial images (fast spin-echo sequences: repetition time, 400.0 ms; echo time, 6.7 ms; layer thickness 4.0 mm; layer spacing 1.0 mm), T1-weighted sagittal images (fast spin-echo sequences: repetition time, 580.0 ms; echo time, 7.5 ms; layer thickness 4.0 mm, layer spacing 0.5 mm), T2-weighted axial images (fast spin-echo sequences: repetition time, 5600.0 ms; echo time, 85.0 ms; layer thickness 4.0 mm; layer spacing 1.0 mm) and T2-weighted coronal images (fast spin-echo sequences: repetition time, 3020.0 ms; echo time, 102.0 ms; layer thickness 4.0 mm; layer spacing 1.0 mm) were obtained. T1-weighted fat-suppressed images in axial and coronal plane orientation were obtained after the intravenous injection of 0.1 mmol/kg body weight gadolinium acid Portuguese amine (Gd-DTPA; Beilu Pharmaceutical, Beijing, China). Images of diffusion-weighted magnetic resonance imaging (DWI) were acquired with b values of 0 and 800 s/mm^2^.

### Image assessment

All images were independently reviewed by two radiologists and a clinician, each with more than 10 years’ experience in head and neck cancer and all blinded to the patients’ clinical status. Disagreements were resolved by consensus. In L-M CT scoring system [25], each paranasal sinus (anterior ethmoid, posterior ethmoid, maxillary, frontal, and sphenoid sinus on the left and right sides) was assigned a point of 0, 1 or 2 according to the severity of inflammation. A similar MRI scoring system was established following this L-M CT scoring system. In the MRI scoring system, a score of 0 indicates that mucosal thickness is less than 3 mm and without sinus opacities, 1 indicates that mucosal thickness is equal or greater than 3 mm and with local soft-tissue shadow or effusion in paranasal sinus, and 2 indicates complete opacification of the sinus. The total score was obtained by summing up score of each sinus on both sides. Since the common loss of the scan of ostiomeatal complex (OMC) in MRI and the inclusion of OMC in L-M CT scoring system, for standardized comparison, the final score in MRI scoring system was equal to the original score scaling up by a factor of 12/10 following the published literature [26]. Whether there is sinusitis and the severity of sinusitis were determined based on the final total score. According to the report of Ashraf N et al [26, 27], corrected total score ≤ 4 was used as the threshold for the absence of sinusitis (no sinusitis in clinical diagnosis). Therefore, categorized by MRI score before RT, group A and B were defined as group with sinusitis (n = 156) and without sinusitis (n = 190), respectively.

### Patient follow-up and statistical analysis

Complete pre-treatment and relatively complete follow-up MRI data was available in all patients. Patients were followed up at least every 3 months in the first year after treatment and every 6 months thereafter. Routine follow-up examinations included a complete head and neck examination, hematological and biochemical examination, chest X-ray and abdominal ultrasonography. Follow-up nasopharynx and neck MRI was performed every 3 to 6 months, especially for patients with suspicion of NPC recurrence or complications caused by RT. Sinus status of patients was evaluated by MRI before RT and 1, 3, 6, 9, 12, 18 and 24 months after RT. Due to the compliance of patients and the disease conditions judged by oncologists, not every patient completed the MRI examination after RT at all follow-up time points.

The clinical features and MRI findings of sinusitis in patients with NPC were compared and analyzed. As the presence of sinusitis before RT may influence the judgement of the final outcome, the risk factors for development of RIS were analyzed in 190 patients without sinusitis before RT (L-M score ≤ 4). A cut-off point at 50 years old was used to divide the age of patients. The counting data were expressed by frequency and percentage (%), and categorical variables were expressed with the measurement of absolute number and percentage (%). Subgroup analyses were performed using Chi-square test. Differences of L-M score between the 2 groups with or without sinusitis before RT at various time points were evaluated by generalized estimation equation. Binary logistic regression analysis was used to establish a model to predict the risk of RIS in NPC patients. Model performance was assessed by the Hosmer-Lemeshow goodness-of-fit test and area under the receiver operating characteristic (ROC) curve. Statistical analyses were performed by using SPSS version 26 software (SPSS, Inc., Chicago, IL, USA) and R software (version 4.1.3), and *p* values < 0.05 were considered statistically significant.

## Result

### Clinical characteristics of NPC patients before RT

The median age of this study population was 48 years (range from 23 to 76 years) and out of these, the male/female ratio was 2.93:1 (258 males, 88 females). As mentioned above, patients were divided into group A (156 patients) and group B (190 patients) according whether with sinusitis before RT. There was no statistically significant difference between the two groups in terms of age, gender, pathological type, N-staging, M-staging, smoking history, intranasal medication, nasal irrigation, RT modality and chemotherapy modality whereas there were statistically differences in T-staging, TNM staging, dose of primary lesions, nasal cavity/paranasal sinus invasion between two groups (Table 1). Baseline characteristics of the two groups were listed in Table 1.

**Table 1:** Clinicopathological features of 346 NPC patients before RT

Characteristics	Group A (with sinusitis before RT)(N/%)	Group B (without sinusitis before RT)(N/%)	χ^2^	*p*
**Age (years)**			0.226	0.634
≤ 50	88(56.4%)	112(58.9%)		
>50	68(43.6%)	78 (41.1%)		
**Gender**			0.441	0.507
Male	119(76.3%)	139(73.2%)		
Female	37(23.7%)	51(26.8%)		
**Pathological type**			1.477	0.224
Non-keratinizing squamous cell carcinoma	148(94.8%)	185(97.4%)		
Keratinizing squamous cell carcinoma	8(5.1%)	5(2.6%)		
Basaloid squamous cell carcinoma	0(0.0%)	0(0.0%)		
T-staging*			64.074	0.000
T1	7(4.5%)	26(13.7%)		
T2	32(20.5%)	78(41.1%)		
T3	50(32.1%)	71(37.4%)		
T4	67(42.9%)	15(7.9%)		
**N-staging**			4.793	0.188
N0	10(6.4%)	24(12.6%)		
N1	34(21.8%)	47(24.7%)		
N2	78(50.0%)	84(44.2%)		
N3	34(21.8%)	35(18.4%)		
**M-staging**			0.500	0.480
M0	144(92.3%)	179(94.2%)		
M1	12(7.7%)	11(5.8%)		
TNM staging*			36.008	0.000
I	1(0.6%)	5(2.6%)		
II	17(10.9%)	36(18.9%)		
III	44(28.2%)	95(50.0%)		
IV	94(60.3%)	54(28.4%)		
**Smoking history**			0.040	0.841
Yes	69(44.2%)	82(43.2%)		
No	87(55.8%)	108(56.8%)		
**Intranasal medication**			0.784	0.376
Yes	73(46.8%)	98(51.6%)		
No	83(53.2%)	92(48.4%)		
**Nasal irrigation**			0.857	0.355
Yes	148(94.9%)	184(96.8%)		
No	8(5.1%)	6(3.2%)		
**Radiotherapy modality**			2.609	0.106
3D-CRT	58(37.2%)	87(45.8%)		
HT	98(62.8%)	103(54.2%)		
Dose of primary lesions (Gy)*			4.376	0.036
≤ 70	143(91.7%)	160(84.2%)		
>70	13(8.3%)	30(15.8%)		
**Chemotherapy modality**			3.791	0.285
None	7(4.5%)	3(1.6%)		
concurrent chemotherapy	15(9.6%)	25(13.2%)		
Induction & concurrent chemotherapy	74(47.4%)	95(50.0%)		
Induction & concurrent & adjuvant/consolidation chemotherapy	60(38.5%)	67(35.3%)		
Nasal cavity/paranasal sinus invasion*			59.417	0.000
Yes	73(46.8%)	19(10.0%)		
No	83(53.2%)	171(90.0%)		

* The differences between group A and group B were statistically significant (*P* < 0.05)

### Changes in MRI-based L-M score

**Fig. 1:**
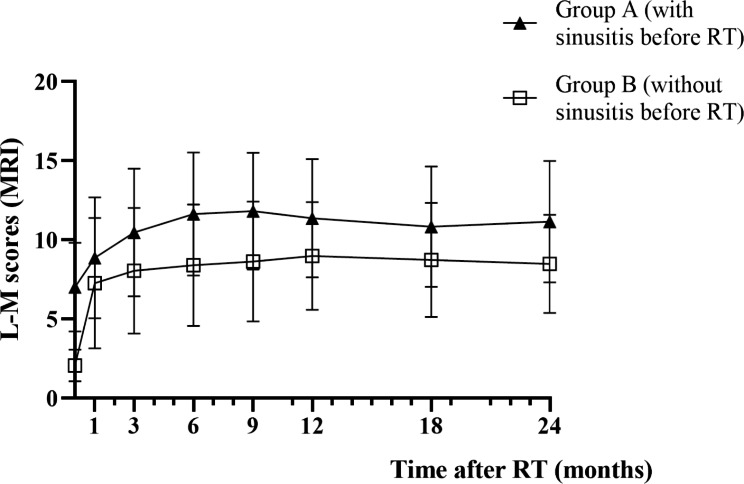
Changes of L-M score (MRI) in NPC patients before and after RT

The mean MRI-based L-M scores in group A and group B before RT were 7.03 ± 2.808 and 2.08 ± 1.002 respectively, and changed to 11.15 ± 3.835 and 8.49 ± 3.105 24 months after RT. The L-M scores of NPC patients in both groups exhibited a rising trend after RT, and reached the highest value 6 months after RT and then tended to plateau (Fig. 1). It was revealed a significant difference in L-M scores at different time point before and after RT in both groups (group A: *p* = 0.000, group B: *p* = 0.000) by repeated measure analysis of variance. However, no statistically significant change of L-M scores from 6 months to 2 years after RT was found in the two groups by further analysis (group A: *p* = 0.311, group B: *p* = 0.469), which indicated that the L-M scores of the two groups tended to be stabilized in 6 months after RT.

The L-M scores of NPC patients in both group A and group B exhibited a rising trend after RT, and reached the highest value 6 months after RT and then tended to plateau.

### Prevalence of RIS

**Fig. 2:**
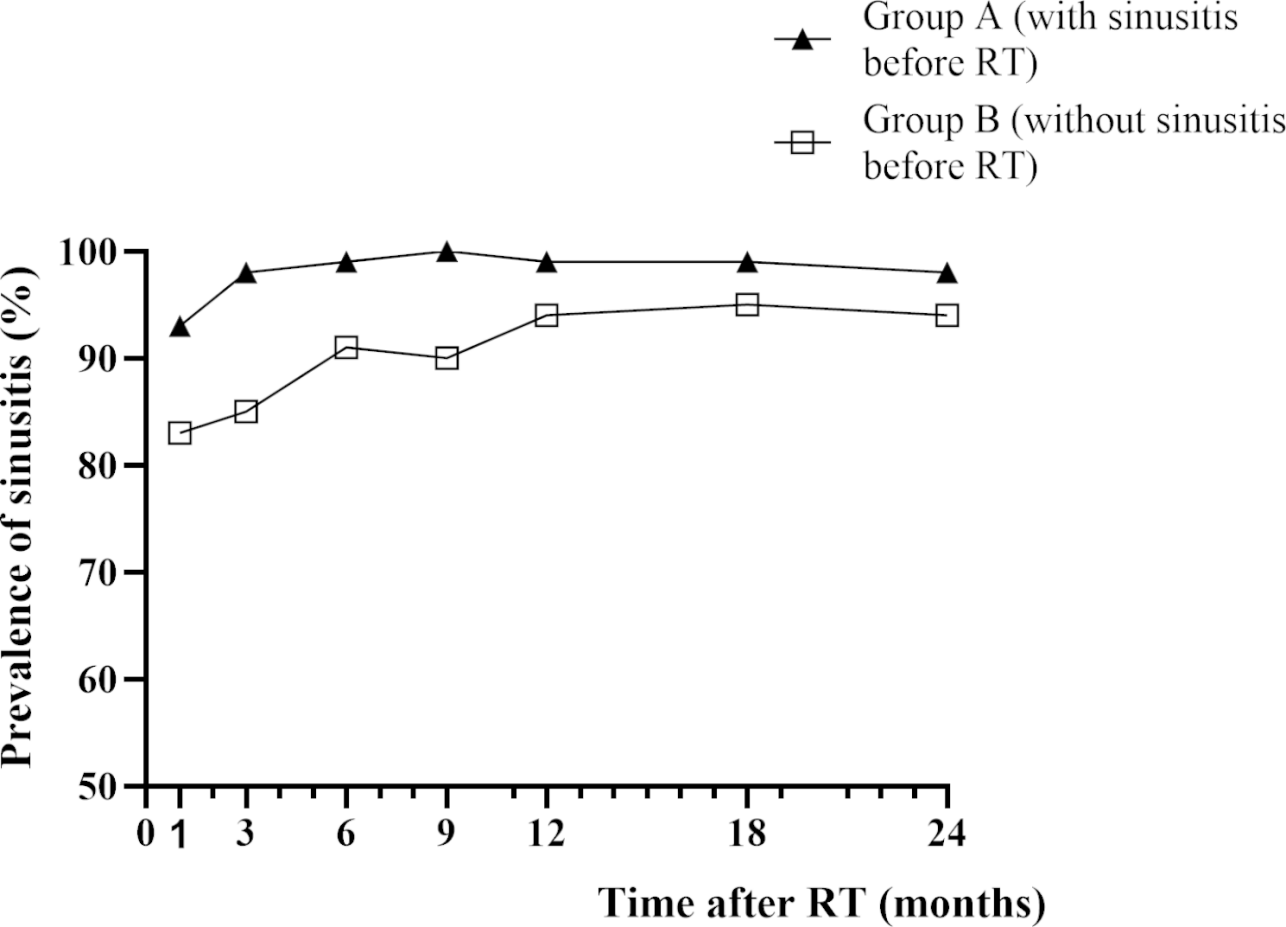
The Changes of RIS prevalence during 24 months follow-up time in two groups

Based on the corrected L-M total score of 4 or higher as the diagnostic criterion for sinusitis, the prevalence of sinusitis in group A and group B at 1, 3, 6, 9 and 12 months after RT was 93% vs. 83%, 98% vs. 85%, 99% vs. 91%, 100% vs. 90%, 99% vs. 94%, respectively. Among the patients without sinusitis before RT (group B), most of the patients exhibited MRI manifestations of rhinosinusitis 1 month after RT (83.2%), the prevalence of sinusitis reached a peak at 6 months after RT (91.2%), and there were still MRI manifestations of sinusitis 2 years after RT (94.0%). For group A, the proportion of patients with MRI findings of rhinosinusitis showed a downward trend within 1 month after RT (93.1%), and gradually increased to 6 months (99.0%) and then tended to be stable. Most patients in group A still had the MRI findings of sinusitis 2 years after RT (98.3%) (Fig. 2).

### Prevalence of sinusitis in different sinuses after RT

**Fig. 3:**
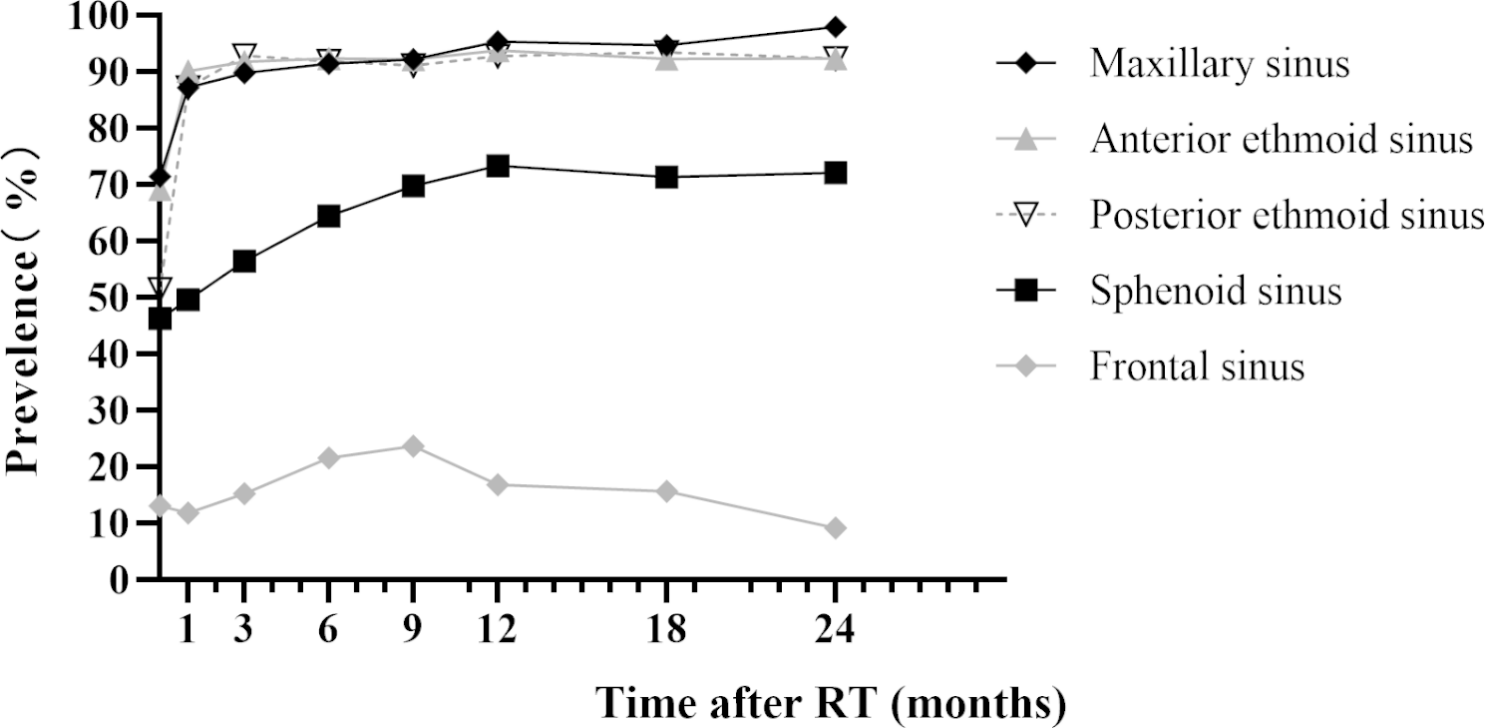
The prevalence of different sinus inflammation in all NPC patients based on MRI findings

The prevalence of RIS in maxillary sinus and ethmoid sinus was the highest in all NPC patients (maxillary sinus: 97.9%, anterior ethmoid sinus: 92.3%, posterior ethmoid sinus: 92.3%) 24 months after RT, followed by sphenoid sinus (72%) and finally frontal sinus (9.1%). The trend of the prevalence of RIS based on MRI findings in other followed time were similar, with the highest prevalence of maxillary sinus and ethmoid sinus, followed by sphenoid sinus and then frontal sinus. In addition, the MRI manifestations of maxillary sinus and ethmoid sinus were found in most patients 1 month after RT (maxillary sinus: 87.1%, anterior ethmoid sinus: 90.0%, posterior ethmoid sinus: 87.1%, sphenoid sinus: 49.5%, frontal sinus: 11.8%). The prevalence of inflammation in sphenoid sinus gradually increased to stable level within 1 year after RT, while that in frontal sinuses decreased gradually after reaching the peak at 9 months after RT (Fig. 3).

### Risk factors for RIS

The following parameters were included as variables for univariate analysis (Table 2): time (1, 3, 6, 9, 12, 18 and 24 months after RT), age (≤ 50 vs. > 50 years), gender (male vs. female), pathological type (non-keratinizing squamous cell carcinoma vs. keratinizing squamous cell carcinoma), T-staging (T1, T2, T3, T4), N-staging (N1, N2, N3, N4), M-staging (M0 vs. M1), TNM staging (I, II, III, IV), smoking history (yes vs. no), intranasal medication (yes vs. no), nasal irrigation (yes vs. no), radiotherapy modality (3D-CRT vs. HT), dose of RT for primary lesions (≤ 70 vs. >70 Gy), chemotherapy modality (none, concurrent chemotherapy, induction & concurrent chemotherapy, induction & concurrent & adjuvant/consolidation chemotherapy), nasal cavity/paranasal sinus invasion (yes vs. no). The results showed that the time, smoking history and TNM staging were associated with the occurrence of RIS.

**Table 2:** Univariate logistic regression analysis of RIS in 190 NPC patients without sinusitis before RT

Variables	*p*	*OR*	95%*CI*
Time (months post-RT for NPC)	0.012		
3 months after RT	0.025	0.318	0.117 ~ 0.865
6 months after RT	0.052	0.367	0.134 ~ 1.009
9 months after RT	0.455	0.662	0.225 ~ 1.953
12 months after RT	0.296	0.551	0.181 ~ 1.684
18 months after RT	0.878	1.100	0.324 ~ 3.738
24months after RT	0.787	1.192	0.333 ~ 4.269
Age	0.703	0.914	0.578 ~ 1.447
Gender	0.141	0.701	0.437 ~ 1.125
Pathological type	0.271	0.538	0.179 ~ 1.621
T-staging	0.306		
T2	0.557	1.221	0.627 ~ 2.377
T3	0.386	1.353	0.683 ~ 2.680
T4	0.380	0.689	0.300 ~ 1.584
N-staging	0.229		
N2	0.241	1.641	0.717 ~ 3.759
N3	0.982	1.008	0.497 ~ 2.044
N4	0.513	0.770	0.352 ~ 1.686
M-staging	0.114	0.526	0.237 ~ 1.167
TNM staging	0.085		
II	0.591	1.439	0.381 ~ 5.432
III	0.474	1.584	0.449 ~ 5.592
IV	0.788	0.841	0.238 ~ 2.969
Smoking history	0.000	2.806	1.651 ~ 4.771
Intranasal medication	0.735	0.925	0.590 ~ 1.452
Nasal irrigation	0.178	0.253	0.034 ~ 1.873
Radiotherapy modality	0.279	1.282	0.818 ~ 2.011
Dose of primary lesions	0.491	1.263	0.650 ~ 2.452
Chemotherapy modality	0.480		
Concurrent chemotherapy	0.492	0.476	0.057 ~ 3.957
Induction & concurrent chemotherapy	0.828	0.795	0.101 ~ 6.277
Induction & concurrent & adjuvant/consolidation chemotherapy	0.762	0.726	0.091 ~ 5.773
Nasal cavity/paranasal sinus invasion	0.816	1.095	0.509 ~ 2.357

Univariate logistic regression analysis of RIS in 190 NPC patients without sinusitis before RT. It showed that the time, smoking history and TNM staging were associated with the occurrence of RIS (*p <* 0.1)

### Multivariate analysis of influencing factors for RIS

**Fig. 4:**
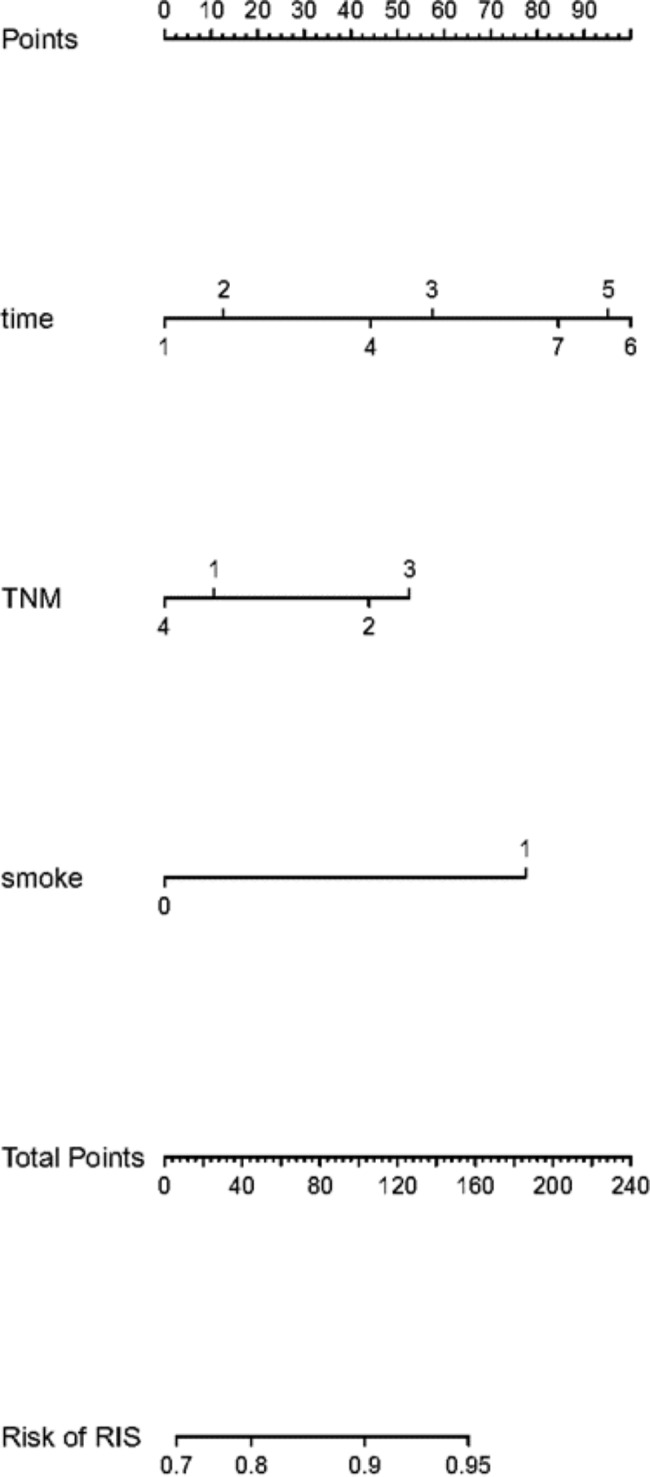
Prognostic nomogram for RIS of NPC patients Time = post-RT months for NPC (1 = 1 month, 2 = 3 months, 3 = 6 months, 4 = 9 months, 5 = 12 months, 6 = 18 months, 7 = 24 months), TNM = clinical stage of NPC (1 = stage I, 2 = stage II, 3 = stage III, 4 = stage IV), smoke = smoking history (0 = no, 1 = yes)

**Fig. 5:**
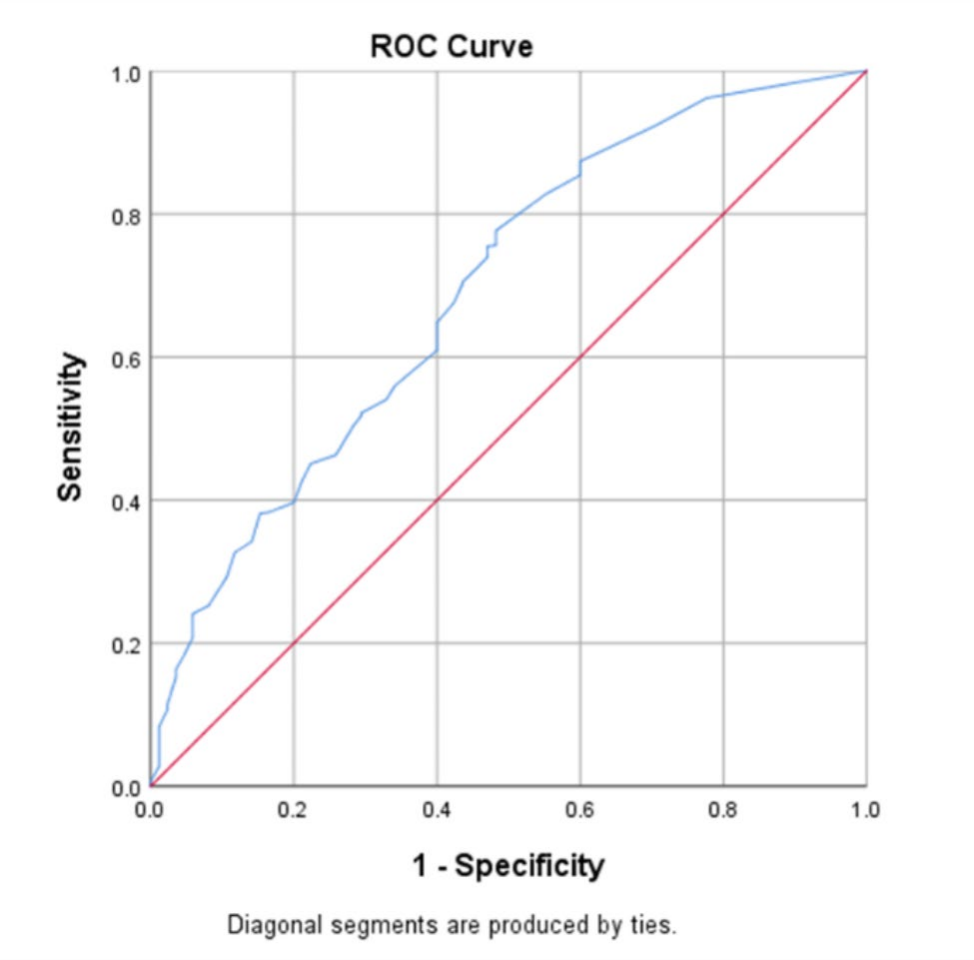
ROC curve for the logistic regression model in group B It showed that the model had a modest discriminatory ability (AUC = 0.692, 95%*CI* = 0.631–0.753, *p* < 0.05)

After forward inclusion of significant variables in univariate analysis, smoking history (*p* < 0.001), time (months post-RT for NPC) (*p* < 0.008) and TNM staging (*p* < 0.040) were found to be independent prognostic factor of RIS in NPC patients (Table 3). According to these above results, a logistics regression model was constructed as “y = a + b + c + 0.909” (Fig. 4). The value of “a” depended on the months after RT and was assigned as 0, 0.176, 0.801, 0.617, 1.328, 1.395, 1.179 when months after RT was 1, 3, 6, 9, 12, 18, 24, respectively. Besides, the value of “b” was decided by the TNM stage and was assigned as 0, 0.464, 0.584, 0.148 when TNM staging was stage I, II, III and IV, respectively. Moreover, the value of “c” depended on the smoking history and was assigned as 0 or 1.081 without or with smoking history. The sensitivity of this model was 77.7% while the specificity was 51.8%. There was no significant difference between the actual and predicted values (*χ*^*2*^ = 7.289, df = 8, *p* = 0.506) by Hosmer and Lemeshow tests. Correspondingly, the constructed ROC curve showed that the model had a modest discriminatory ability (AUC = 0.692, 95%*CI* = 0.631–0.753, *p* < 0.05) (Fig. 5).

**Table 3:** Multivariate logistic regression analysis of RIS in 190 NPC patients without sinusitis before RT.

Variables	*p*	*OR*	95%*CI*
Time (months post-RT for NPC)	0.008		
3 months after RT	0.589	1.193	0.629 ~ 2.262
6 months after RT	0.035	2.229	1.057 ~ 4.701
9 months after RT	0.131	1.854	0.831 ~ 4.136
12 months after RT	0.006	3.773	1.470 ~ 9.687
18 months after RT	0.007	4.036	1.472 ~ 11.068
24 months after RT	0.023	3.250	1.180 ~ 8.955
TNM staging	0.040		
II	0.506	1.591	0.405 ~ 6.253
III	0.379	1.794	0.488 ~ 6.590
IV	0.823	0.862	0.235 ~ 3.167
Smoking history	0.000	2.949	1.721 ~ 5.052
constant	0.177	2.483	

## Discussion

Data on the prevalence of sinusitis in NPC patients is relatively deficient, and there is also a relative paucity of research on these patients with RT of 3D-CRT or HT. It is reasonable and necessary to investigate the prevalence and risk factors of RIS in NPC patients. Although CT examination is currently considered as popular imaging standard for the diagnosis and evaluation of sinusitis [28, 29], in clinical practice, the routine imaging examination of NPC after RT is prone to be cranial MRI rather than CT, and less paranasal sinus CT. Therefore, there is a relative lack of data on the prevalence of sinusitis based on MRI in NPC patients after RT. Nevertheless, using MRI to evaluate sinusitis in NPC patients after RT is very practical for clinical diagnosis and treatment. Our study demonstrated that sinusitis in NPC patients after RT was time-specific and sinus-specific, and also exhibited the correlation between the occurrence of RIS and some clinical characteristics (time, smoking history, TNM staging). Accordingly, based on our research data, we constructed a prediction model with certain diagnostic ability.

### Superiority and shortcomings of MRI in the evaluation of RIS

Lin and Bhattacharyya et al. reported that when using L-M scoring system to evaluate manifestations of CT and MRI on paranasal sinuses, there was no significant difference between the average L-M scores based on MRI and CT [23]. Consequently, it should be feasible to evaluate the RIS using MRI instead of CT. Besides, CT imaging cannot precisely identify the abnormal components in paranasal sinus, while MRI scanning can achieve better visualization of soft tissues and distinguish thickened mucosa, secretion retention, concomitant retention cysts and tumors accurately. The surveillance of tumor in NPC patients was usually conducted by MRI, so the judgment of RIS based on MRI may be not only more in line with the actual situation of clinic, but also better understand the changing dynamics of sinus inflammation without additional examination. As reported by Brenner et al., repeated CT scans increased the risk of cancer [30]. Furthermore, compared with CT, extra radiation exposure is avoided in using MRI [31]. Therefore, it may be unnecessary, costly and even harmful to perform extra sinus CT in NPC patients who have already undergone MRI.

Meanwhile, there are also some defects in the imaging diagnosis of RIS using MRI. MRI is inferior to CT in distinguishing skeletal anatomy. MRI examination requires long examination time and is performed in a relatively cramped and small environment, so it may require to use sedative for people with claustrophobia. Moreover, MRI may be overly sensitive to the evaluation of mucosal thickening, and some patients with simple sinus mucosal thickening may be asymptomatic. The features mentioned above are required to pay close attention to when MRI is used to evaluate sinusitis after RT [32].

### The development of RIS

Similar to the results of most previous studies evaluated by CT [29, 33], the MRI-based L-M scores of NPC patients without sinusitis before RT was significantly increased after RT. These patients generally had MRI manifestations of sinusitis at 1 month after RT, and the incidence reached its highest peak at 6 months after RT, and then gradually stabilized. MRI findings of sinusitis could still be found in NPC patients 2 years after RT. These results indicated that the sinus opacification of NPC patients after RT is directly related to RT, and the damage of nasal mucosa does not stop after the termination of RT, and forward progresses within half a year after RT. This persistent damage would be mainly related to the delayed effect of radiation, as shown in the study of Yin et al. [34]. The effect of radiation damage is persistent after RT so the impact of RT on paranasal sinus mucosa is significant and lasting long time. In this study, most patients (97.12%) received different forms of chemotherapy before or at the same time with RT. The damage of human immune system caused by chemotherapy which led to the decrease of pathogen resistance of nasal and paranasal sinus mucosa may aggravate sinusitis after RT. However, in our study, there was no significant association between chemotherapy and RIS, which might due to the too small sample proportion of patients without chemotherapy (3 cases / 1.6%).

Our study showed that ethmoid sinus and maxillary sinus were the most vulnerable sinuses for therapeutic toxicity after RT in NPC patients, similar to the reports of Huang and Hsin [35, 36], even if the radiation dose of ethmoid sinus and maxillary sinus was not as high as that of sphenoid sinus which was closer to the primary tumor. The difference in the prevalence of RIS in various sinuses may attribute to the anatomical characteristics of each sinus, such as the position of the sinus opening, the adjacent relationship of each sinus, and different mucociliary clearance (MCC). The openings of the maxillary sinus and ethmoid sinus are likely blocked since they are in the lowest and middle parts of the semilunar hiatus of relatively narrow OMC with complicated structure. For its large cavity and upper maxillary sinus ostium, the maxillary sinus is relatively difficult to drain. Impaired sinus drainage is also prone to occur in the ethmoid sinus with honeycomb structure, small and numerous air chambers with small drainage port when the sinus mucosa swells after infection. Owing to the proximity to the nasopharynx, sphenoid sinus is the most commonly invaded sinus. But the incidence of sinusitis in sphenoid sinus is even lower than that in other sinuses. This may due to the dramatic shrinkage of the tumor after RT and the sphenoid sinus ostium situated in the backmost part of nasal cavity alone. Besides these above anatomical factors, the impairment of sinus function is also an important factor leading to RIS. The study of Surico et al. showed that children who received head RT had lower nasal MCC rate and longer mucociliary transport time than those who did not treated with head RT, suggesting that RT may lead to impaired mucociliary function, and even permanent damage. Morphological and ultrastructural studies revealed that there was extensive tissue destruction in sinonasal mucosa [37]. In addition, RT may also cause the squamous metaplasia and subepithelial edema of nasal and paranasal sinus mucosa [38]. The research of Kamel et al. demonstrated that the delay of MCC affected the anterior group (excluding the frontal sinus) much more than the posterior group of sinuses [29]. It should also increase the incidence of inflammation in the maxillary sinus and ethmoidal sinus after RT.

### Risk factors for RIS

#### Post radiotherapy time

Similar to other researches, our study showed that post RT time was one of the independent influencing factors for RIS [29, 33, 39]. Kamel et al. reported that effects on MCC persist after RT [29].The post-RT MCC delay time gradually deteriorated over the following 6 months after RT, and then stabilized, and persisted. The recovery of mucosal function usually did not depend on the time after RT, as the damage may be permanent. The ultrastructural damage of nasal mucosal epithelium caused by RT can be still detectable more than 20 years later [37]. RIS presented acute inflammation in the early stage, such as edema of nasal mucosa, increase of nasal discharge, inhibition of ciliary excretion function and retention of secretions. When the radiation dose increased to 60–70 Gy, necrosis and sloughing of the sinonasal mucosa would occur, aggravating the retention of secretion of the paranasal sinuses. Irreversible mucosal atrophy and distortion would occur in the late stage, such as nasal adhesions, choanal stenosis, and/or atresia [34, 37, 39–41]. The irreversible damage of ciliary function of sinonasal mucosa in NPC patients leads to sinus drainage impairment, secretion retention and accumulation, which makes it easier for the penetration of bacteria into the sinuses. After bacteria invade the sinuses, MCC delay time in NPC patients gradually worsen with time, and the incidence and severity of RIS gradually increased concomitantly [29, 42].

### Smoking history

Resembling the study of Chi-Che Huang [35], our multivariate analysis indicated that smoking history was also independently associated with the development of RIS in NPC patients. Tobacco smoke extracts had caused adverse effects on MCC of nasal mucosal epithelium, innate immune function and olfactory mucosal metaplasia [43]. Smoking may promote the development of RIS by affecting the pathophysiological function of sinonasal mucosa. The research of Hannah N showed that there were greater squamous metaplastic changes and subepithelial edema in RIS compared with chronic rhinosinusitis without nasal polyps [38]. These mucosal changes of squamous metaplasia were similar to nasal metaplasia caused by toxic damage of inhalant exposure (such as smoking). Consequently, smoking may aggravate the metaplastic changes and tissue remodeling of nasal mucosa caused by radiation.

### TNM staging

In our study, we demonstrated that the occurrence of RIS was related to TNM staging. The view of most previous studies was that the later period of the tumor-related stage, the higher the incidence of RIS [35, 39, 44]. In this predictive model, TNM stage II and TNM stage III were risk factors for RIS, suggesting that the clinical staging of NPC was related to the development of RIS. However, TNM stage IV was a protective factor for RIS in this model. The reason may be that there were some selection biases in the included patients. According to statistical design, only patients without sinusitis before RT were included in the univariate and multivariate analyses of risk factors for sinusitis, while most of the patients with stage IV were excluded since they already suffered from rhinosinusitis before RT (63.5%, Table 1). The high incidence of sinusitis in stage IV NPC patients before RT may due to tumors invasion into the nasal cavity and sinuses and choanal obstruction, which seriously affecting the ventilation and drainage of the sinuses. Therefore, the proportion of stage IV patients decreased within the study population, which was similar to other studies [39]. In addition, the patients of stage IV included in this study mainly had the tumor invading the brain, hypopharynx, orbit, parotid gland and other tissues, and less invasion of the sinuses and nasal cavity (only 10% of the patients without sinusitis before RT in this study had sinonasal invasion). The selection bias may contribute mostly to the reason that stage IV was a protective factor for RIS.

#### Radiotherapy modality

Our study revealed that there was no significant relationship between two conformal radiotherapy modalities in the occurrence of RIS, which may be attributed to the following two points. Firstly, compared to other RT modality, HT is mainly aimed at protecting the important tissues and organs adjacent to NPC, such as brainstem, eyeball, cranial nerve, temporomandibular joint, and so on. However, when outlining the range of RT, there is no targeted protection for the paranasal sinuses which account for a large proportion of the skull and its functions are relatively less important. Therefore, there may be no significant difference in the radiation dose for paranasal sinuses between HT and 3D-CRT. Secondly, the anatomical characteristics of the sinuses have a great impact on the occurrence of sinusitis after RT, while the anatomical variations of the paranasal sinuses were similar in the patients with different radiotherapy modalities. However, our results are not consistent with those of Pei-Wen et al., which may require further prospective studies to confirm [45].

### Other factors

Nasal irrigation and intranasal medication are the primary treatments for RIS, which can clean nasal cavity, enhance mucociliary function, and improve local inflammation. Some studies have shown that long-term nasal irrigation is beneficial for improving the quality of life [46] and nasal symptoms [47] of NPC patients after RT. However, for these patients, there was no significant difference in imaging changes of post-RT sinusitis between NPC patients with or without nasal irrigation [47]. FENG et al. demonstrated that patients who received fluticasone propionate aqueous nasal spray combined with nasal irrigation had fewer nasal complaints, a better life quality and lower endoscopy scores than those who received only nasal irrigation within 6 months after RT, but neither therapy (fluticasone propionate aqueous nasal spray combined with nasal irrigation, or nasal irrigation alone) changed the CT scores of post-RT sinusitis [48]. These results were similar to our study.

However, in this study, criteria of nasal irrigation and medication included not only long-term regular use, but also treatments only during RT, which may affect the statistical results. In addition, the dependent variable in this study is the qualitative indicator for whether there are radiographic signs of sinusitis, which can not reveal the severity of sinusitis. Whether the nasal irrigation and intranasal medication improve the imaging manifestation needed further research to draw a clear conclusion.

High-dose radiation may cause a permanent decline in nasal mucociliary function [37]. Radiation of 40 Gy or more can cause acute mucosal inflammation, while the radiation of 60 ~ 70 Gy could cause ischemic mucosal necrosis and mucosal sloughing [33]. There was less damage to the transfer function of the nasal mucociliary system, when the irradiation was less than 37 Gy [16, 34]. Nevertheless, the NPC patients involved in this study were treated with a relatively small range of variation local RT dose of 64 to 76 Gy, which may be a crucial factor that the radiation dose did not affect the occurrence of RIS in this study.

Different from our results, Su’s study showed that, the incidence of sinusitis in NPC patients after IMRT was positively correlated with T stage and nasal invasion [39]. The possible reason may due to the difference of the percentage of people with nasal cavity/paranasal sinus invasion in those patients without sinusitis before RT. For the NPC patients without sinusitis before RT, only 10% of them had nasal cavity/sinuses invasion, while in the Su’s study, the percentage was much higher as 58.7%.

#### Prediction model

Different from other multivariate analyses of RIS [35, 36, 39, 44], we constructed the prediction model and evaluated its predictive power. However, this model cannot predict the severity of RIS and may not be helpful since 90% and more of NPC patients have sinusitis after RT. For all that, the identified predictors may help to guide further counselling and management for the clinic.

#### Summary

This study was based on the MRI scoring system to study the progression, characteristics and risk factors of sinusitis in patients with NPC after 3D-CRT or HT, and developed a predictive model of RIS. At present, few researches in this aspect were reported. Our study is not only innovative but also of great significance in clinical practice, for it presented a certain reference value for the treatment and prevention of RIS. Our results indicate that MRI can also make a good judgment on the status of sinusitis and can be applied to evaluate RIS of NPC patients. However, there were still some limitations since it was a single-center retrospective analysis, the number of patients was relatively small and the bias of persuasion by the most experienced member of the decision-making committee. A multi-center, large-sample, randomized and prospective study is needed to further verify these conclusions.

## Data Availability

Data supporting the conclusions of this article are available from the corresponding author.

## Abbreviations

MRI: Magnetic resonance imaging
NPC: Nasopharyngeal carcinoma
RIS: Radiation-induced sinusitis
RT: Radiotherapy
L-M: Lund-Mackay
AUC: Area under the curve
HT: Helical tomotherapy
3D-CRT: Three-dimensional conformal radiation therapy
2D-RT: Two-dimensional radiation therapy
IMRT: Intensity-modulated radiation therapy
SRT: Stereotactic radiation therapy
IGRT: Image-guided radiation therapy
DGRT: Dose-guided radiation therapy
CRT: Chemoradiotherapy
CT: Computer tomography
UICC/AJCC: Union for International Cancer Control/American Joint Committee on Cancer
GTV: Gross tumor volume
CTV: Clinical target area
CI: conformity index
HI: homogeneity index
OMC: Ostiomeatal complex
ROC: Receiver operating characteristic
MCC: Mucociliary clearance

## Declarations

### Study subjects or cohorts overlap

No study subjects or cohorts have been previously reported.
